# Real-World Treatment Outcomes After Nivolumab Progression in BRAF-Negative Metastatic Melanoma: A Multicenter Cohort Study by the Turkish Oncology Group

**DOI:** 10.3390/jcm15135224

**Published:** 2026-07-03

**Authors:** Emine Bihter Eniseler, Atike Pinar Erdogan, Mustafa Şahbazlar, Fatma Keskin Uzundere, Teoman Şakalar, Hasibe Bilge Gür, İlhan Hacıbekiroğlu, Onur Yazdan Balcık, İsmail Beypınar, Mehmet Gürdal Savsar, Gözde Pempe, Sila Oksuz, Tuğba Başoğlu, Özge Demirkıran, Bilgin Demir, Bedriye Açıkgöz Yıldız, Atike Gökçen Demiray, Mehmet Sinan Akarca, İlkay Tuğba Ünek, Mahmut Kara, Muslih Urun, Ahmet Cebeli Gökay, Havva Yeşil, Ferhat Ekinci

**Affiliations:** 1 Department of Medical Oncology, Manisa Celal Bayar University, Manisa 45030, Turkey; 2 Department of Medical Oncology, Dicle University, Diyarbakir 21280, Turkey; 3 Department of Medical Oncology, Kahramanmaraş Sütçü İmam University, Kahramanmaraş 46050, Turkey; 4 Department of Medical Oncology, Sakarya University, Adapazari 54100, Turkey; 5 Department of Medical Oncology, Alanya Alaaddin Keykubat Üniversitesi, Alanya 07425, Turkey; 6 Department of Medical Oncology, Mersin Training and Research Hospital, Mersin 33000, Turkey; dr.gurdalsavsar@hotmail.com (M.G.S.);; 7 Department of Medical Oncology, Dr. Lütfi Kırdar Kartal Eğitim ve Araştırma Hastanesi, Istanbul 34865, Turkey; 8 Department of Medical Oncology, Aydin Adnan Menderes University, Aydin 09100, Turkey; 9 Depatment of Medical Oncology, Pamukkale University, Denizli 20160, Turkey; bedriyeacikgoz09@gmail.com (B.A.Y.);; 10 Department of Medical Oncology, Dokuz Eylül University, Izmir 35330, Turkey; 11 Department of Medical Oncology, Van Yuzuncu Yil University, Van 65090, Turkeymuslihurun@gmail.com (M.U.); 12 Department of Medical Oncology, Gaziantep University, Gaziantep 27310, Turkey

**Keywords:** metastatic melanoma, nivolumab, immune checkpoint inhibitors, chemotherapy, anti-PD-1 progression

## Abstract

**Background/Objectives:** Despite improved survival with immune checkpoint inhibitors, the optimal treatment after anti-PD-1 progression in metastatic melanoma remains unclear. This study compared survival outcomes and treatment responses between chemotherapy (CT)- and immunotherapy (IO)-based therapies administered after nivolumab progression in patients with BRAF-negative metastatic melanoma. **Methods:** This multicenter retrospective study included patients with BRAF-negative metastatic melanoma who developed disease progression during nivolumab treatment. Post-progression systemic therapies were categorized as CT- or IO-based treatments. Treatment responses were assessed according to RECIST version 1.1 criteria. Progression-free survival (PFS) and overall survival (OS) were analyzed using the Kaplan–Meier method, and prognostic factors were evaluated using Cox regression analyses. **Results:** A total of 141 patients were included. Following nivolumab progression, 107 (75.9%) received CT and 34 (24.1%) received IO. Based on best response to nivolumab, the objective response rate (ORR; CR + PR) was 55.1% in the CT group and 44.1% in the IO group. After post-nivolumab treatment, ORRs were 29.9% and 32.4% in the CT and IO groups, respectively, whereas complete response rates were higher with IO (21.2% vs. 3.0%). Median PFS was 4.17 months in the CT group and 3.9 months in the IO group (*p* = 0.403). Median OS was 7.83 and 8.17 months, respectively (*p* = 0.416). Elevated LDH level was identified as an independent adverse prognostic factor. **Conclusions:** In this multicenter real-world cohort, no statistically significant differences in survival were observed between patients receiving CT or IO after nivolumab progression. Given the retrospective, non-randomized study design, these findings should not be interpreted as evidence of comparative treatment efficacy. The higher CR rate observed with IO should be interpreted cautiously due to potential selection bias. Prospective studies are warranted to define the optimal treatment strategy after anti-PD-1 failure.

## 1. Introduction

Melanoma, although relatively uncommon among skin cancers, is one of the leading causes of skin cancer–related mortality due to its aggressive biological behavior and high metastatic potential [1]. Once metastatic disease develops, prognosis markedly worsens, and systemic therapy becomes the cornerstone of treatment in most patients with advanced melanoma. The metastatic behavior of melanoma is shaped by the interaction of multiple biological mechanisms, including tumor biology, the immune microenvironment, and metabolic heterogeneity [2,3]. Therefore, systemic therapies play a central role in the management of advanced melanoma.

The introduction of immune checkpoint inhibitors into clinical practice has led to a major paradigm shift in the treatment of metastatic melanoma. Owing to the survival benefits achieved with programmed death-1 (PD-1) inhibitors, immunotherapy (IO) has become one of the cornerstone treatment modalities in advanced melanoma [4,5,6]. Current clinical guidelines recommend nivolumab, pembrolizumab, the nivolumab–relatlimab combination, and the nivolumab–ipilimumab combination among first-line treatment options for metastatic or unresectable melanoma [7].

Despite the clinical benefits achieved with PD-1 inhibitors, a substantial proportion of patients experience disease progression during or after treatment. The management of metastatic melanoma patients progressing after anti-PD-1 therapy remains a significant challenge in clinical practice. Although current guidelines recommend various IO combinations or alternative systemic treatment options in this setting, the level of evidence regarding subsequent-line treatment strategies remains limited, and treatment decisions are often based on individualized clinical assessment [7,8,9].

In particular, patients with BRAF-negative metastatic melanoma represent a clinically important subgroup in terms of treatment sequencing. Since BRAF/MEK-targeted therapies are not applicable in this population, post-progression treatment options are largely limited to IO or cytotoxic chemotherapy (CT) [9,10]. Studies evaluating the efficacy of CT following immune checkpoint inhibitor treatment have demonstrated that clinical responses may still be achieved in selected patients, although outcomes remain heterogeneous overall [11,12].

Real-world data suggest that treatment patterns following IO may be more heterogeneous than those reported in clinical trials. These studies provide important insights into the effectiveness and survival outcomes of therapies administered after anti-PD-1 treatment [13,14]. Nevertheless, data regarding clinical outcomes of subsequent-line treatments following nivolumab progression remain limited.

In this study, we aimed to evaluate real-world outcomes of subsequent systemic treatment approaches in patients with BRAF-negative metastatic melanoma who developed disease progression following nivolumab treatment. In this multicenter study conducted across Türkiye, post-nivolumab treatment modalities, treatment responses, treatment-related adverse events, and survival outcomes were analyzed.

## 2. Materials and Methods

### 2.1. Study Design and Patient Population

This retrospective multicenter cohort study included patients with histopathologically confirmed metastatic melanoma who were treated with nivolumab for metastatic or unresectable disease between January 2015 and December 2024 at 12 oncology centers in Türkiye and had confirmed BRAF mutation-negative status based on institutional molecular pathology methods.

The study population consisted of patients who developed radiologically confirmed disease progression during or after nivolumab treatment and subsequently received at least one line of systemic therapy following progression. Patients with incomplete clinical or laboratory data, as well as those who did not receive any systemic treatment after nivolumab progression, were excluded from the study.

### 2.2. BRAF Status Assessment

BRAF mutation status was assessed using institutional molecular pathology methods routinely employed in clinical practice at participating centers. BRAF-negative status was determined based on molecular pathology reports documented in patient records. Although testing methods varied across centers, polymerase chain reaction (PCR)-based methods, targeted molecular analyses, or next-generation sequencing (NGS) techniques were used in appropriate cases.

### 2.3. Data Collection

Demographic, clinical, and laboratory data of the patients were retrospectively obtained from the electronic medical record systems of the participating centers. Data collection was performed using a standardized data collection template across all centers.

Collected variables included age, sex, ECOG performance status, smoking history, disease stage at diagnosis, metastatic status, metastatic sites, and comorbidities. Laboratory parameters recorded during the metastatic disease period included hemoglobin, neutrophil, lymphocyte, platelet count, lactate dehydrogenase (LDH), alkaline phosphatase (ALP), alanine aminotransferase (ALT), C-reactive protein (CRP), and serum albumin levels.

Treatment-related data included the best response to nivolumab treatment, the type of systemic therapy administered after nivolumab progression, treatment-related adverse events, and post-progression treatment responses.

### 2.4. Post-Progression Treatment Groups

Systemic treatments administered following nivolumab progression were categorized into two main groups according to treatment modality: CT and IO-based treatments. Treatment classification was based on the first systemic therapy administered after nivolumab progression.

The CT group included cytotoxic regimens such as dacarbazine, temozolomide, paclitaxel, carboplatin–paclitaxel combination, and cisplatin/vinblastine/dacarbazine (CVD). Less frequently used CT protocols were classified as “other CT regimens.”

The IO group included immune checkpoint inhibitor–based treatments such as ipilimumab plus nivolumab combination therapy, pembrolizumab monotherapy, ipilimumab monotherapy, pembrolizumab combined with low-dose ipilimumab, and nivolumab plus relatlimab combination therapy.

Treatment allocation was not randomized and was determined according to routine clinical practice at participating centers, taking into account clinical evaluation, patient characteristics, performance status, prior response to nivolumab, treatment accessibility, and reimbursement conditions.

### 2.5. Response Evaluation

Treatment responses were assessed according to the Response Evaluation Criteria in Solid Tumors (RECIST) version 1.1 criteria [15]. Best treatment response was classified as complete response (CR), partial response (PR), stable disease (SD), or progressive disease (PD). The objective response rate (ORR) was calculated as the sum of CR and PR rates.

Radiological response assessments were performed by responsible investigators at each participating center, primarily based on contrast-enhanced computed tomography (CT) imaging. Positron emission tomography/computed tomography (PET/CT) findings were considered as supportive assessments when clinically indicated.

The best response to nivolumab treatment and the best response to therapies administered after nivolumab progression were evaluated separately.

Due to the retrospective nature of the study, iRECIST or immune-related response criteria for the assessment of IO-specific atypical response patterns were not routinely used. Therefore, immune-related atypical response patterns, such as pseudoprogression, may not have been fully evaluated.

### 2.6. Outcome Measures

Progression-free survival (PFS) was defined as the time from the initiation of treatment administered after nivolumab progression to the date of radiologically confirmed disease progression or death from any cause.

Overall survival (OS) was calculated as the time from the initiation of treatment administered after nivolumab progression to death from any cause.

Patients without progression or death were censored at the date of last follow-up. Follow-up duration was calculated from the initiation of post-progression treatment to the date of the last clinical assessment or death. Follow-up evaluations and radiological imaging were performed according to routine clinical practice at participating centers.

### 2.7. Statistical Analysis

Statistical analyses were performed using IBM SPSS Statistics version 26.0 (IBM Corp., Armonk, NY, USA). The distribution of continuous variables was assessed using skewness–kurtosis measures and the Kolmogorov–Smirnov test. Variables with skewness and kurtosis values between −3 and +3 and showing no significant deviation from normality in the Kolmogorov–Smirnov test were considered normally distributed. Normally distributed continuous variables were presented as mean ± standard deviation, whereas non-normally distributed variables were reported as median (minimum–maximum) or median (interquartile range [IQR]), as appropriate.

Comparisons of continuous variables between two groups were performed using Student’s *t*-test or the Mann–Whitney *U* test according to data distribution. Categorical variables were expressed as counts and percentages and compared using the chi-square test or Fisher’s exact test.

Survival analyses were performed using the Kaplan–Meier method, and differences between groups were assessed using the log-rank test. Univariable and multivariable Cox proportional hazards regression analyses were performed to identify prognostic factors associated with OS and PFS.

Considering the limited sample size and number of events, a parsimonious modeling approach was adopted to minimize the risk of overfitting in multivariable models. Accordingly, variables with a *p*-value < 0.10 in univariable analyses were included in multivariable models. Results were reported as hazard ratios (HRs) with 95% confidence intervals (CIs).

A two-sided *p*-value < 0.05 was considered statistically significant for all analyses.

### 2.8. Ethical Approval

This study was initially designed as a single-center retrospective study, and the relevant ethical approval was obtained accordingly. Following the expansion of the study into a nationwide multicenter cohort, the study protocol was re-evaluated and approved as a multicenter study by the Health Sciences Ethics Committee of Manisa Celal Bayar University Faculty of Medicine (Decision Date/No: 29 April 2026/20.478.486/6101).

The study was conducted in accordance with the principles of the Declaration of Helsinki. Due to the retrospective nature of the study, the requirement for informed consent was waived by the ethics committee.

## 3. Results

### 3.1. Patient Characteristics

A total of 181 patients with BRAF-negative metastatic melanoma were initially assessed. After excluding patients with incomplete clinical/laboratory data or those who did not receive any systemic treatment following nivolumab progression, a total of 141 patients were included in the study. Among patients who developed disease progression during or after nivolumab treatment, 107 (75.9%) received CT, whereas 34 (24.1%) received IO after progression. The median follow-up duration was 16.4 months (range, 1.8–75.6 months). The demographic and clinical characteristics of the patients are presented in Table 1.

No statistically significant differences were observed between treatment groups in terms of age and the majority of laboratory parameters measured during the metastatic disease period (*p* > 0.05). However, serum albumin levels differed significantly between the groups (*p* < 0.001). No significant differences were observed in hemoglobin, neutrophil, lymphocyte, platelet count, LDH, ALP, ALT, or CRP levels between the treatment groups (all *p* > 0.05).

Among categorical variables, smoking status was significantly associated with the type of treatment administered after nivolumab progression (*p* = 0.038). In the CT group, 51.4% of patients were never smokers, 14.0% were current smokers, and 34.6% were former smokers. Corresponding proportions in the IO group were 67.6%, 20.6%, and 11.8%, respectively.

The presence of diabetes mellitus (DM) was also significantly associated with treatment type (*p* = 0.015). The prevalence of DM was 23.4% in the CT group and 47.1% in the IO group. No significant differences were observed between treatment groups with respect to ECOG performance status, stage at diagnosis, metastatic status, metastatic sites, primary tumor type, other comorbidities, or NGS testing status.

### 3.2. Treatment Responses

A statistically significant association was observed between the best response to nivolumab treatment and the type of subsequent treatment administered (*p* < 0.001). In the CT group, the distribution of best response to nivolumab treatment was 6.5% for complete response (CR), 48.6% for partial response (PR), 13.1% for stable disease (SD), and 31.8% for progressive disease (PD). Corresponding rates in the IO group were 29.4%, 14.7%, 17.6%, and 38.2%, respectively (Table 2). Accordingly, the objective response rate (ORR) to nivolumab treatment was 55.1% in the CT group and 44.1% in the IO group.

When best responses to treatments administered after nivolumab progression were evaluated, a statistically significant difference was also observed between treatment groups (*p* = 0.002). In the CT group, CR, PR, SD, and PD rates were 2.8%, 28.0%, 22.4%, and 46.7%, respectively, whereas corresponding rates in the IO group were 20.6%, 11.8%, 17.6%, and 50.0%, respectively (Table 2). The ORR to post-nivolumab treatment was 30.8% in the CT group and 32.4% in the IO group.

### 3.3. Treatment-Related Adverse Events

The distribution of treatment-related adverse events revealed differences in the frequency of certain toxicities between treatment groups. Rash was observed more frequently in the IO group compared with the CT group (32.4% vs. 12.1%, *p* = 0.014). Similarly, elevated liver enzymes were more common in the IO group (23.5% vs. 9.3%, *p* = 0.031). A significant difference was also observed in the incidence of hypothyroidism, which occurred in 38.2% of patients in the IO group compared with 2.8% in the CT group (*p* < 0.001). No significant differences were observed between treatment groups for other adverse events.

Myelosuppression, defined as treatment-related anemia, neutropenia, and/or thrombocytopenia, was observed in 55 of 107 patients (51.4%) in the CT group, including grade 3–4 events in 8 patients (7.5%).

Grade ≥ 3 adverse events were uncommon in both treatment groups. In the CT group, the most frequent grade ≥ 3 toxicities were nausea (6.5%) and fatigue (3.7%), whereas loss of appetite (8.8%) and fatigue (5.9%) were the most common grade ≥ 3 toxicities in the IO group. No significant differences were observed between treatment groups regarding the frequency of severe adverse events (Table 3).

### 3.4. Survival Analyses

**Figure 1:**
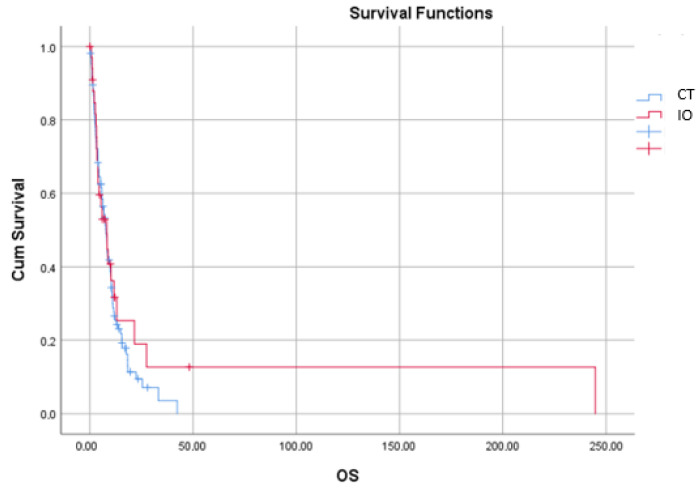
Kaplan–Meier Overall Survival Curves According to Treatment Group (CT vs. IO).

**Figure 2:**
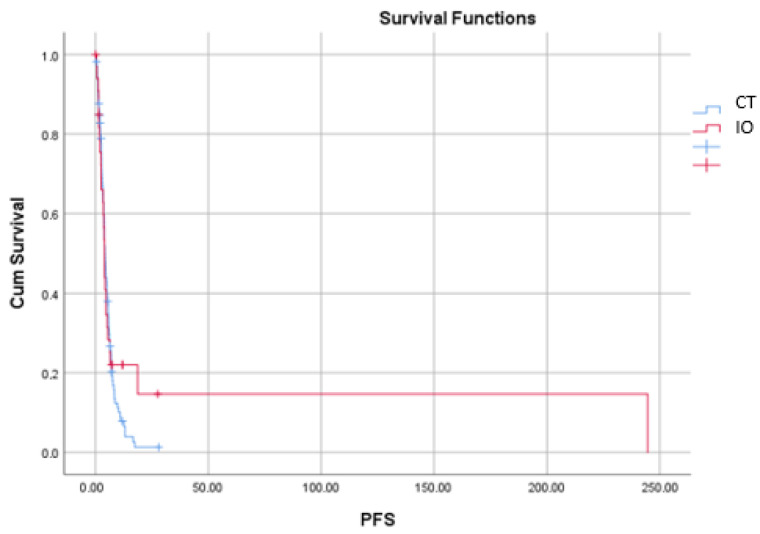
Kaplan–Meier Progression-Free Survival Curves According to Treatment Group (CT vs. IO).

Kaplan–Meier analysis demonstrated no statistically significant differences in PFS or OS according to the type of treatment administered after nivolumab progression. Median PFS was 4.17 months (95% CI: 3.575–4.765) in the CT group and 3.9 months (95% CI: 3.446–4.354) in the IO group (log-rank *p* = 0.403). Median OS was 7.83 months (95% CI: 6.153–9.507) in the CT group and 8.17 months (95% CI: 3.663–12.677) in the IO group, with no statistically significant difference observed between the groups (log-rank *p* = 0.416). PFS and OS curves according to treatment groups are presented in Figure 1 and Figure 2, respectively.

Subgroup analyses demonstrated significant associations between certain clinical variables and survival outcomes (Table 4). In the IO group, the presence of bone metastasis was associated with poorer survival outcomes in terms of both OS and PFS. Median OS was 8.8 months in patients without bone metastasis and 3.27 months in those with bone metastasis (*p* = 0.006). Similarly, median PFS was 4.67 months in patients without bone metastasis compared with 1.43 months in those with bone metastasis (*p* = 0.002).

In the CT group, the presence of lung metastasis was significantly associated with PFS. Median PFS was 3.83 months in patients with lung metastasis and 5.07 months in those without lung metastasis (*p* = 0.031).

No significant associations were observed between survival outcomes and stage at diagnosis, metastatic status, brain metastasis, liver metastasis, tumor localization, or best response to nivolumab treatment (Table 4).

### 3.5. Cox Regression Analyses

In the CT group, univariable analysis of OS demonstrated that LDH levels at the time of metastasis were significantly associated with an increased risk of mortality (HR = 1.35; 95% CI: 1.13–1.62; *p* = 0.001). In multivariable analysis, LDH remained an independent prognostic factor for OS (HR = 1.36; 95% CI: 1.11–1.68; *p* = 0.004). In addition, NGS testing status was statistically associated with OS in both univariable analysis (HR = 2.20; 95% CI: 1.37–3.54; *p* = 0.001) and multivariable analysis (HR = 3.12; 95% CI: 1.90–5.12; *p* < 0.001) (Table 5). However, this finding should be interpreted as a healthcare-process-related association rather than evidence that NGS testing itself represents an intrinsic biological prognostic factor.

In the CT group, age, LDH level at the time of metastasis, and the presence of lung metastasis were significantly associated with PFS in univariable analyses. However, none of these variables retained independent prognostic significance in multivariable analysis (Table 6).

In the IO group, LDH level at the time of metastasis was identified as an independent prognostic factor for both OS and PFS. In multivariable analysis, elevated LDH levels were associated with an increased risk of mortality (HR = 1.50; 95% CI: 1.01–2.24; *p* = 0.045). Similarly, elevated LDH remained an independent predictor of progression in the PFS analysis (HR = 1.81; 95% CI: 1.18–2.77; *p* = 0.006). Although bone metastasis and NGS status were significant in univariable analyses, they did not retain independent prognostic significance in multivariable analysis (Table 5 and Table 6).

## 4. Discussion

In this multicenter real-world study, survival outcomes of CT- and IO-based treatments administered after nivolumab progression were compared in patients with BRAF-negative metastatic melanoma who developed disease progression during nivolumab treatment. The principal finding of our study was the absence of a statistically significant difference in PFS and OS according to the type of treatment administered following nivolumab progression. Nevertheless, the markedly higher CR rate observed in patients receiving IO was an unexpected finding that warrants careful interpretation. Taken together, these observations suggest that treatment selection after anti-PD-1 progression is likely influenced not only by treatment modality but also by disease biology and patient-related characteristics.

The 21.2% CR rate observed in patients receiving IO in our study represents a noteworthy finding. Although data regarding IO rechallenge after prior IO remain limited, clinically meaningful responses to subsequent IO have been reported in selected patient populations [16]. This finding suggests that biological factors such as immune microenvironment characteristics and tumor-infiltrating lymphocyte patterns may contribute to renewed treatment responses [16].

However, the unexpectedly high CR rate observed in the IO group should be interpreted with caution. This finding is unlikely to indicate superior efficacy of post-progression IO over CT. Rather, patients selected for subsequent IO had a substantially higher complete response rate during prior nivolumab treatment, suggesting that clinicians preferentially offered further IO to patients who had previously demonstrated sensitivity to immune checkpoint inhibition. Moreover, treatment allocation was influenced by physician judgment, patient characteristics, reimbursement policies, and treatment availability. Together with the relatively small size of the IO group, these factors may have contributed to the observed response rates. Therefore, the higher CR rate should be considered hypothesis-generating rather than evidence of superior treatment efficacy.

Although immune checkpoint inhibitors constitute the backbone of advanced melanoma treatment, the optimal post-progression treatment strategy for patients progressing under anti-PD-1 therapy remains unclear [17,18].

Although no statistically significant differences in PFS or OS were observed between treatment groups in this retrospective cohort, these findings should not be interpreted as evidence of equivalent efficacy between chemotherapy and immunotherapy. Because treatment allocation was not randomized and substantial selection bias may have influenced treatment choice, our results merely describe outcomes observed in routine clinical practice and should be considered exploratory.

In our study, the median OS achieved with post-nivolumab treatments was approximately 8 months. This finding is consistent with previous real-world studies evaluating treatment efficacy after immune checkpoint inhibitor failure. In the multicenter retrospective analysis by Goldinger et al., median OS following CT administered after IO ranged between 6 and 9 months [11]. Similarly, compared with studies reporting median PFS durations of approximately 3–4 months, the median PFS of nearly 4 months observed in our cohort appears consistent with the existing literature [11,13].

Likewise, recently published real-world data have demonstrated that systemic treatments administered after anti-PD-1 therapy generally provide limited but measurable clinical benefit. In a large retrospective analysis by Betof Warner et al. evaluating different systemic treatment sequences after anti-PD-1 failure, median PFS following CT was approximately 3–4 months, whereas median OS ranged between 7 and 9 months [19]. Similarly, Hassel et al. demonstrated that survival outcomes after immune checkpoint inhibitor failure remained limited and were largely influenced by patient selection and disease biology [20].

Although immune checkpoint inhibitors have substantially improved survival in metastatic melanoma, real-world evidence indicates that a considerable proportion of patients still develop disease progression during anti-PD-1 treatment [21].

Current international guidelines emphasize that there is no standard treatment strategy for patients with metastatic melanoma progressing after anti-PD-1 therapy and recommend individualized treatment selection. NCCN and ESMO guidelines suggest consideration of IO combinations, alternative IO strategies, CT, and clinical trial options in eligible patients [8,22]. The absence of a statistically significant survival difference in our cohort is consistent with the uncertainty surrounding optimal treatment selection after anti-PD-1 progression and underscores the need for individualized clinical decision-making.

Current studies suggest that CT administered after IO failure may still provide limited clinical benefit in selected patients [11]. Furthermore, dacarbazine-based regimens administered after IO have been reported to produce longer-than-expected response durations in certain patients [13].

Disease progression in metastatic melanoma is influenced not only by tumor burden but also by tumor metabolism and microenvironmental characteristics. Metabolic heterogeneity and increased glycolytic activity in tumor cells may contribute to metastatic potential [2].

A noteworthy finding of our study was the identification of elevated LDH levels at the time of metastasis as an adverse prognostic factor in both CT and IO groups. In the IO group, LDH retained independent prognostic significance for both OS and PFS, supporting its role as a clinically relevant biomarker in metastatic melanoma. Elevated LDH has consistently been associated with poor treatment response and inferior survival outcomes in patients receiving IO [1].

Another notable finding was the significant difference in baseline serum albumin levels between the treatment groups. Serum albumin is widely recognized as a marker of nutritional status, systemic inflammation, and overall physiological reserve in patients with advanced cancer. Therefore, the observed difference may partly reflect differences in nutritional status between the groups, as suggested by the reviewer. In addition, variations in disease burden, inflammatory activity, and clinician-driven treatment selection may also have contributed to this finding. Because treatment allocation was not randomized, baseline imbalances between groups cannot be excluded. Consequently, this observation should be interpreted cautiously and should not be considered a direct effect of treatment modality.

NGS testing status was associated with survival outcomes in some analyses; however, this finding should be interpreted with caution. NGS testing itself is not an intrinsic biological prognostic factor and may instead reflect differences in referral patterns, access to molecular diagnostics, treatment intensity, physician preference, or healthcare resource utilization. Therefore, this association should not be interpreted as evidence of a direct causal relationship between NGS testing and clinical outcomes.

Similarly, clinical prognostic factors have been shown to significantly influence treatment outcomes in patients with advanced melanoma treated with immune checkpoint inhibitors. A real-world study from Türkiye demonstrated that several clinical prognostic scores were associated with survival outcomes in advanced melanoma patients treated with IO [23,24]. These findings support the notion that clinical outcomes following IO depend not only on the administered treatment but also on the biological characteristics of the disease and clinical risk factors.

Metastatic patterns are also known to have important effects on clinical outcomes. Melanoma may metastasize to different organs with distinct biological behaviors, and the site of metastatic involvement may influence treatment outcomes [3]. In our study, the presence of bone metastasis in the IO group was associated with poorer OS and PFS outcomes. This finding supports the potential influence of metastatic involvement patterns on IO outcomes.

One of the major strengths of this study is the inclusion of a multicenter real-world cohort involving a large number of institutions across Türkiye. The heterogeneous patient population increases the applicability of our findings to routine clinical practice. Moreover, given the limited data regarding post-nivolumab treatment strategies in patients with BRAF-negative metastatic melanoma, our findings contribute valuable evidence to the existing literature.

Similarly, real-world evidence suggests that treatment patterns and clinical outcomes may be influenced by healthcare system conditions, patient characteristics, and treatment accessibility. In a multicenter real-world study conducted by the Turkish Oncology Group on patients with metastatic uveal melanoma, treatment choices were found to be heterogeneous, and survival outcomes were influenced by both clinical and systemic factors [25]. Although that study included patients with uveal melanoma, its findings highlight the complexity of metastatic melanoma treatment in real-world settings and emphasize that outcomes may depend not only on the administered therapy but also on patient- and healthcare system–related factors.

Although the role of CT has diminished in the IO era, it remains an accepted treatment option for selected patients according to current clinical guidelines, particularly after immune checkpoint inhibitor failure [8,10]. In our cohort, no statistically significant survival difference was observed between treatment groups; however, this finding should not be interpreted as evidence of equivalent treatment efficacy because of the retrospective, non-randomized study design.

On the other hand, dermatologic and endocrine toxicities related to IO are frequently encountered in clinical practice. The higher incidence of hypothyroidism and dermatologic toxicities observed in the IO group in our study is consistent with previously reported immune-related adverse events in the literature [26,27]. Some studies have also suggested that these immune-related toxicities may be associated with treatment efficacy [28].

This study has several important limitations. First, the retrospective study design may have introduced potential sources of bias related to patient selection and treatment preferences. In addition, the limited number of patients within treatment groups may have reduced the statistical power, particularly for subgroup analyses. Because the IO group included only 34 patients, further stratified analyses according to additional clinical characteristics were not performed, as such analyses would have been underpowered and could have produced unreliable estimates with an increased risk of type II error. Therefore, our findings should be interpreted with caution, and larger prospective studies are warranted to validate these results and allow more robust subgroup analyses.

The non-randomized nature of treatment allocation and the influence of patient characteristics, performance status, and physician judgment may have contributed to selection bias. Therefore, both the higher complete response rate observed in the IO group and the survival outcomes should be interpreted with caution.

Furthermore, reimbursement policies and treatment accessibility specific to the healthcare system in which the study was conducted may have influenced treatment selection. In particular, the availability of IO and other systemic treatment options within reimbursement frameworks may have shaped clinical decision-making. This may have contributed to heterogeneity in treatment distribution and may limit the generalizability of the findings to countries with different healthcare systems.

Additionally, the heterogeneity of treatment sequences administered after nivolumab progression may have limited direct comparisons of treatment efficacy. Owing to the retrospective multicenter design, variations in BRAF assessment methods, radiological follow-up intervals, and clinical monitoring practices across participating centers may also have influenced the study findings.

## 5. Conclusions

In conclusion, this multicenter real-world study describes treatment patterns and outcomes in patients with BRAF-negative metastatic melanoma after nivolumab progression. Although no statistically significant differences in survival were observed between patients receiving CT or IO, the non-randomized retrospective design precludes conclusions regarding comparative treatment efficacy. The higher complete response rate observed with IO should be interpreted cautiously due to possible selection bias. Larger prospective studies are needed to define the optimal treatment strategy after anti-PD-1 failure.

## Figures and Tables

**Table 1:** Baseline Demographic and Clinical Characteristics of Patients According to Post-Nivolumab Treatment.

Variable	Category	CT, *n* (%) (*n* = 107)	IO, *n* (%) (*n* = 34)	Total, *n* (%) (*n* = 141)	*p*-Value
Sex	Female	47 (43.9)	13 (38.2)	60 (42.6)	0.700
	Male	60 (56.1)	21 (61.8)	81 (57.4)	
ECOG-PS	0	30 (28.0)	8 (23.5)	38 (27.0)	0.795
	1	65 (60.7)	21 (61.8)	86 (61.0)	
	2–3	12 (11.2)	5 (14.7)	17 (12.1)	
Smoking status	Never	55 (51.4)	23 (67.6)	78 (55.3)	0.038
	Current	15 (14.0)	7 (20.6)	22 (15.6)	
	Former	37 (34.6)	4 (11.8)	41 (29.1)	
Stage at diagnosis	I	2 (1.9)	1 (2.9)	3 (2.1)	0.958
	II	25 (23.4)	9 (26.5)	34 (24.1)	
	III	26 (24.3)	8 (23.5)	34 (24.1)	
	IV	54 (50.5)	16 (47.1)	70 (49.6)	
Metastatic status	De novo	53 (49.5)	13 (38.2)	66 (46.8)	0.341
	Recurrent	54 (50.5)	21 (61.8)	75 (53.2)	
Metastatic sites	Brain	19 (17.8)	4 (11.8)	23 (16.3)	0.577
	Liver	41 (38.3)	9 (26.5)	50 (35.5)	0.293
	Bone	36 (33.6)	8 (23.5)	44 (31.2)	0.370
	Lung	64 (59.8)	15 (44.1)	79 (56.0)	0.159
	Other	59 (55.1)	17 (50.0)	76 (53.9)	0.744
Primary tumor type	Cutaneous	24 (22.4)	8 (23.5)	32 (22.7)	1.000
	Non-cutaneous	83 (77.6)	26 (76.5)	109 (77.3)	
Comorbidities	DM	25 (23.4)	16 (47.1)	41 (29.1)	0.015
	CAD	28 (26.2)	13 (38.2)	41 (29.1)	0.257
	CLD	9 (8.4)	4 (11.8)	13 (9.2)	0.556
NGS testing	Performed	69 (64.5)	16 (47.1)	85 (60.3)	0.108
	Not performed	38 (35.5)	18 (52.9)	56 (39.7)	

Abbreviations: CT, Chemotherapy; IO, Immunotherapy; ECOG-PS, Eastern Cooperative Oncology Group Performance Status; DM, diabetes mellitus; CAD, coronary artery disease; CLD, chronic lung disease.

**Table 2:** Best treatment responses to nivolumab and post-nivolumab therapies according to treatment group.

Response	CT, *n* (%)	IO, *n* (%)	*p*-Value
Best response to nivolumab			<0.001
CR	7 (6.5)	10 (29.4)	
PR	52 (48.6)	5 (14.7)	
SD	14 (13.1)	6 (17.6)	
PD	34 (31.8)	13 (38.2)	
Best response to post-nivolumab therapy			0.002
CR	3 (2.8)	7 (20.6)	
PR	30 (28.0)	4 (11.8)	
SD	24 (22.4)	6 (17.6)	
PD	50 (46.7)	17 (50.0)	

Abbreviations: CT, Chemotherapy; IO, Immunotherapy; CR, complete response; PR, partial response; SD, stable disease; PD, progressive disease.

**Table 3:** Treatment-related adverse events according to post-nivolumab therapy.

Adverse Event	CT, *n* (%)	IO, *n* (%)	Grade ≥ 3 CT, *n* (%)	Grade ≥ 3 IO, *n* (%)	*p*-Value
Diarrhea	10 (9.3)	5 (14.7)	0 (0)	0 (0)	0.377
Rash	13 (12.1)	11 (32.4)	0 (0)	0 (0)	0.014
Fatigue	55 (51.4)	11 (32.4)	4 (3.7)	2 (5.9)	0.082
Nausea	49 (45.8)	11 (32.4)	7 (6.5)	0 (0)	0.237
Loss of appetite	51 (47.7)	13 (38.2)	3 (2.8)	3 (8.8)	0.445
Elevated liver enzymes	10 (9.3)	8 (23.5)	1 (0.9)	1 (2.9)	0.031
Hypothyroidism	3 (2.8)	13 (38.2)	0 (0)	0 (0)	<0.001
Colitis	6 (5.6)	2 (5.9)	0 (0)	0 (0)	1.000
Arthralgia	10 (9.3)	7 (20.6)	0 (0)	1 (2.9)	0.147
Dyspnea	2 (1.9)	3 (8.8)	0 (0)	0 (0)	0.168
Headache	5 (4.7)	2 (5.9)	0 (0)	0 (0)	0.777
Myelosuppression *	55 (51.4)	17 (50.0)	8 (7.5)	1 (2.9)	0.638

Abbreviations: CT, Chemotherapy; IO, Immunotherapy. * Defined as treatment-related anemia, neutropenia, and/or thrombocytopenia.

**Table 4:** Subgroup analyses of overall survival and progression-free survival according to post-nivolumab treatment.

Variable		CT				IO			
		OS (95% CI)	*p*-Value	PFS (95% CI)	*p*-Value	OS (95% CI)	*p*-Value	PFS (95% CI)	*p*-Value
Stage at diagnosis	1–2	10.10 (6.95–13.25)	0.110	4.43 (2.91–5.95)	0.681	13.03 (8.42–17.64)	0.284	6.33 (3.23–9.43)	0.086
	3	8.67 (3.31–14.03)		4.17 (3.37–4.97)		3.47 (1.77–5.18)		2.43 (0.15–4.71)	
	4	6.40 (4.93–7.87)		4.00 (2.99–5.01)		5.47 (0–11.97)		3.40 (2.73–4.07)	
Metastasis status	De novo	6.87 (4.73–9.01)	0.166	4.37 (3.06–5.68)	0.776	5.47 (0.63–10.31)	0.203	3.40 (2.06–4.74)	0.734
	Recurrent	8.40 (6.72–10.08)		4.13 (3.46–4.80)		11.73 (2.75–20.71)		4.00 (3.55–4.45)	
Brain metastasis	No	7.83 (5.82–9.84)	0.547	4.37 (3.53–5.21)	0.462	8.37 (4.37–12.37)	0.120	4.00 (3.45–4.55)	0.609
	Yes	7.07 (1.06–13.09)		2.60 (1.79–3.41)		2.20 (0–4.89)		1.90 (0–4.67)	
Liver metastasis	No	7.83 (4.91–10.75)	0.418	4.30 (3.26–5.34)	0.176	8.17 (4.19–12.15)	0.811	3.90 (3.54–4.26)	0.674
	Yes	7.67 (4.41–10.94)		3.90 (2.85–4.95)		3.27 (2.48–4.06)		2.53 (0.69–4.37)	
Bone metastasis	No	8.13 (6.75–9.51)	0.764	4.00 (3.54–4.46)	0.595	8.80 (5.02–12.58)	0.006	4.67 (3.20–6.14)	0.002
	Yes	6.87 (3.31–10.43)		5.00 (3.58–6.42)		3.27 (2.62–3.92)		1.43 (0–3.00)	
Lung metastasis	No	10.30 (9.50–11.10)	0.051	5.07 (3.48–6.66)	0.031	8.37 (4.69–12.06)	0.080	4.63 (3.54–5.73)	0.098
	Yes	5.90 (4.40–7.40)		3.83 (3.26–4.40)		4.50 (2.02–6.98)		3.33 (2.14–4.53)	
Other metastasis	No	8.23 (6.41–10.05)	0.651	3.77 (2.55–4.99)	0.335	3.90 (3.10–4.70)	0.039	3.33 (2.12–4.54)	0.253
	Yes	7.67 (5.43–9.91)		4.37 (3.07–5.67)		10.30 (4.95–15.65)		4.67 (3.15–6.19)	
Tumor localization	Cutaneous	7.07 (5.04–9.10)	0.192	4.23 (3.69–4.77)	0.587	8.37 (3.56–13.18)	0.700	3.90 (3.36–4.44)	0.365
	Non-cutaneous	9.87 (6.84–12.90)		3.90 (2.39–5.41)		6.07 (0–12.96)		3.90	
Best response to nivolumab	CR	5.77 (1.23–10.31)	0.053	5.07 (2.32–7.82)	0.077	21.67 (8.08–35.27)	0.197	5.43 (2.86–8.00)	0.243
	PR	7.67 (5.75–9.59)		5.07 (3.20–6.94)		4.50 (2.96–6.04)		3.67 (2.17–5.17)	
	SD	11.57 (7.54–15.60)		4.43 (3.46–5.40)		8.37 (0.73–16.01)		4.20 (0.40–8.00)	
	PD	4.50 (2.49–6.51)		3.10 (2.31–3.89)		3.33 (2.19–4.47)		2.53 (0.26–4.80)	

Abbreviations: CT, Chemotherapy; IO, Immunotherapy; OS, overall survival; PFS, progression-free survival; CI, confidence interval; CR, complete response; PR, partial response; SD, stable disease; PD, progressive disease.

**Table 5:** Cox regression analyses of overall survival according to post-nivolumab treatment group.

Variable	CT				IO			
	Univ HR (95% CI)	*p*-Value	Multiv HR (95% CI)	*p*-Value	Univ HR (95% CI)	*p*-Value	Multiv HR (95% CI)	*p*-Value
Age	0.99 (0.97–1.00)	0.13	–	–	1.01 (0.98–1.05)	0.44	–	–
LDH at metastasis	1.35 (1.13–1.62)	0.001	1.36 (1.11–1.68)	0.004	1.68 (1.13–2.52)	0.01	1.50 (1.01–2.24)	0.045
NGS status	2.20 (1.37–3.54)	0.001	3.12 (1.90–5.12)	<0.001	3.10 (1.24–7.76)	0.02	2.02 (0.54–7.51)	0.29

Abbreviations: CT, Chemotherapy; IO, Immunotherapy. Variables with *p* < 0.10 in univariable analyses were entered into multivariable Cox regression models.

**Table 6:** Cox regression analyses of progression-free survival according to post-nivolumab treatment group.

Variable	CT				IO			
	Univ HR (95% CI)	*p*-Value	Multiv HR (95% CI)	*p*-Value	Univ HR (95% CI)	*p*-Value	Multiv HR (95% CI)	*p*-Value
Age	0.98 (0.96–1.00)	0.01	0.99 (0.97–1.00)	0.10	1.00 (0.96–1.03)	0.81	–	–
LDH at metastasis	1.33 (1.09–1.62)	0.004	1.18 (0.95–1.47)	0.14	2.05 (1.35–3.12)	0.001	1.81 (1.18–2.77)	0.006
Lung metastasis	1.58 (1.04–2.41)	0.03	1.51 (0.98–2.35)	0.07	–	–	–	–
NGS status	–	–	–	–	2.24 (1.00–5.03)	0.05	1.45 (0.51–4.07)	0.48
Bone metastasis	–	–	–	–	3.90 (1.54–9.86)	0.004	2.53 (0.75–8.56)	0.14

Abbreviations: CT, Chemotherapy; IO, Immunotherapy. Variables with *p* < 0.10 in univariable analyses were entered into multivariable Cox regression models.

## Data Availability

The datasets generated and/or analyzed during the current study are available from the corresponding author on reasonable request.

## Abbreviations

**Abbreviation**	**Definition**
ALP	Alkaline Phosphatase
ALT	Alanine Aminotransferase
BRAF	B-Raf Proto-Oncogene
CAD	Coronary Artery Disease
CI	Confidence Interval
CR	Complete Response
CRP	C-Reactive Protein
CT	Chemotherapy
CTCAE	Common Terminology Criteria for Adverse Events
CVD	Cisplatin/Vinblastine/Dacarbazine
DM	Diabetes Mellitus
ECOG-PS	Eastern Cooperative Oncology Group Performance Status
HR	Hazard Ratio
IO	Immunotherapy
IQR	Interquartile Range
LDH	Lactate Dehydrogenase
NCCN	National Comprehensive Cancer Network
NGS	Next-Generation Sequencing
ORR	Objective Response Rate
OS	Overall Survival
PCR	Polymerase Chain Reaction
PD	Progressive Disease
PD-1	Programmed Death-1
PET/CT	Positron Emission Tomography/Computed Tomography
PFS	Progression-Free Survival
PR	Partial Response
RECIST	Response Evaluation Criteria in Solid Tumors
SD	Stable Disease
TNM	Tumor–Node–Metastasis
